# Study on Antimicrobial Resistance and Molecular Characteristics of *Riemerella anatipestifer*

**DOI:** 10.3390/ani16030442

**Published:** 2026-01-31

**Authors:** Ming Yan, Xiaofei Song, Hui Chen, Hongxue Zhang, Youzhi Li, Daozheng Liu, Baotao Liu, Ming Zou

**Affiliations:** 1 College of Veterinary Medicine, Qingdao Agricultural University, Qingdao 266000, China; m15192379585@163.com; 2 Qilu Animal Health Products Co., Ltd., Jinan 250100, China; 18663761943@163.com (X.S.); 17867112773@163.com (H.C.); hongxue.zhang@163.com (H.Z.); 3 Shandong Province Key Laboratory of Quality Safety Monitoring for Animal Products and Veterinary Drug Innovation, Shandong Center for Quality Control of Feed and Veterinary Drug, Jinan 250100, China; liyouzhi2009@126.com; 4 Qingdao Lijian Biotechnology Co., Ltd., Qingdao 266034, China; 18354258819@163.com

**Keywords:** *Riemerella anatipestifer*, antibiotic-resistance gene, virulence gene, serotype, epidemiology

## Abstract

*Riemerella anatipestifer* (*R. anatipestifer*) is a pathogenic bacterium belonging to the genus *Riemerella* within the family Flavobacteriaceae, which has multidrug resistance (MDR) and can cause high pathogenicity in waterfowl. The aim of this study was to investigate the antimicrobial resistance and genomic characteristics of *R. anatipestifer* strains isolated from several regions of China from 2023 to 2024. Two strains were selected for challenge tests, and virulence protection tests were conducted on florfenicol-resistant and florfenicol-sensitive strains. A total of 88 strains of *R. anatipestifer* were collected from the Shandong, Jiangsu, Guangdong, Hebei and Henan regions. The results showed that the 88 strains included serotypes 1, 2, 5, 6, 7 and 10. Serotype 5 was the most prevalent in the Shandong region. All strains were multidrug-resistant, with the hexaresistance accounting for the highest proportion (42.1%). A total of five resistance genes (*tet(X)*, *floR*, *ermF*, *qnrS*, *rmtB*) and seven virulence genes were found (*ompA*, *camp*, *AS87_04050*, *SIP*, *Fur*, *TbdR1*, *luxE*). The challenge test showed that the LD_50_ of RA12 was 2.75 × 10^7^ CFU/mL, and that of RA26 was 2.57 × 10^7^ CFU/mL. Phylogenetic tree analysis revealed that strain RA26 was closely related to strain 20190403E1-1, and strain RA16 was closely related to strain JW1. In addition, serotypes 2 and 7 identified in this study have been undergoing clonal transmission in China. Virulence protection tests indicated that the results of in vitro drug susceptibility tests were consistent with the therapeutic effects after in vivo treatment, and no *R. anatipestifer* was found in the visceral tissues of surviving ducklings. This study provides a reference for the rational use of antibiotics.

## Simple Summary

At present, the antimicrobial resistance of *Riemerella anatipestifer* has become increasingly severe in China, causing substantial economic losses to the poultry breeding industry. Relevant research reports on this bacterium remain relatively scarce. The aim of this study was to investigate the antimicrobial resistance and genomic characteristics of *R. anatipestifer* strains isolated from several regions of China from 2023 to 2024, and conduct virulence protection tests to verify the in vivo protective effect of effective antimicrobial agents, thereby providing references for the prevalence status and clinical management of *Riemerella anatipestifer* disease.

## 1. Introduction

*Riemerella anatipestifer* (*R. anatipestifer*) is a major pathogenic bacterium of invasive infection, which belongs to the genus *Riemerella* in the family Flavobacteriaceae. *R. anatipestifer* mainly infects 1–8-week-old ducklings, causing serositis and peritonitis, and has caused enormous economic losses in the waterfowl breeding industry [1]. The infection of *R. anatipestifer* was first discovered in 1932 in Long Island, New York [2]. At present, there are a total of 21 serotypes known in international reports [3], with high prevalence and no significant cross-protective effects between each serotype. The dominant prevalent serotypes in China are serotypes 1, 2, 6, 7 and 10 [4], which pose a huge challenge to the development of effective vaccines against this pathogen.

According to research reports, the virulence factors involved in *R. anatipestifer* mainly include *camp* hemolysin [5], *ompA* and putative genes for *LPS* synthesis [6]. Research indicates that *R. anatipestifer* naturally exhibits resistance to multiple classes of antibiotics, including aminoglycosides, tetracyclines, cephalosporins, macrolides, sulfonamides and polymyxins [7]. According to the research report by Liu et al. (2024) on the *R. anatipestifer* strains isolated from Anhui Province, the resistance rates of this pathogen to aminoglycosides (amikacin, neomycin, gentamicin, kanamycin, streptomycin) were all higher than 96%, with a tetracycline resistance rate of 98%, and the resistance rates to fluoroquinolones also exceeded 96% [4]. This indicated a high prevalence of multidrug resistance (MDR) in the *R. anatipestifer* strains prevalent in the Anhui region. Therefore, it is particularly important to clarify the antimicrobial resistance mechanisms of *R. anatipestifer*. The antimicrobial resistance results of 56 Anhui goose-derived strains isolated by Zhang et al. (2024) showed a 100% resistance rate to kanamycin, streptomycin, gentamicin, azithromycin, sulfadimethoxine and sulfamethoxazole, and all these strains were identified as multidrug-resistant strains [8]. The research findings by Yang et al. demonstrated that the strains exhibited high-level resistance to sulfamethoxazole (99.5%), kanamycin (96.2%), gentamicin (93.1%), ofloxacin (92.9%) and trimethoprim (91.4%) [7]. In recent years, the prevalence of antibiotic resistance in *R. anatipestifer* has become extremely severe in China; however, few studies have reported the prevalence and antimicrobial resistance profiles of the strains isolated from Shandong Province. The selection of Shandong Province as the study area is strongly justified by its pivotal role in China’s poultry industry. Official statistics reveal the province’s large-scale and growing poultry sector: in 2024, the number of poultry slaughtered reached 2.50 billion, with poultry meat output amounting to 4.902 million tons. For comparison, the figures for 2023 were 2.38 billion slaughtered and 4.769 million tons of meat produced [Data Source: Shandong Provincial Bureau of Statistics, Statistical Communique of Shandong Province on the 2024 National Economic and Social Development, released 7 May 2025]. Investigating the epidemiological profile and antimicrobial resistance patterns of *R. anatipestifer* in such a high-density, large-scale production area is therefore critical for developing effective disease control strategies. Therefore, this study conducted an epidemiological investigation and antimicrobial resistance analysis on the *R. anatipestifer* strains in Shandong Province. This focus addresses a significant gap in the existing literature concerning this major production hub.

This study aimed to investigate the antimicrobial resistance and genomic characteristics of *R. anatipestifer* strains isolated from different regions of China from 2023 to 2024. Since there were no unified, international antimicrobial resistance breakpoints for *R. anatipestifer*, other relevant studies have also referred to the criteria for *Escherichia coli* ATCC 25922 and *Pasteurella multocida* for interpretation. Therefore, challenge experiments were performed in this study, and drug-resistant and drug-sensitive strains were simultaneously screened for virulence protection tests. The consistency between the in vivo therapeutic effects of the drugs and the in vitro antimicrobial susceptibility test results was observed, which provides a reference for the rational clinical use of drugs in the treatment of *R. anatipestifer* infections.

Notably, compared with recent large-scale epidemiological studies in China (2022–2024), our study provides several novel insights: (1) we successfully isolated and characterized *R. anatipestifer* from chickens (oviduct and embryos), which are rarely reported hosts, expanding the understanding of its host range; (2) beyond in vitro susceptibility profiling, we integrated in vivo virulence protection assays to directly validate the correlation between florfenicol resistance phenotypes and therapeutic outcomes in ducklings, offering stronger evidence for clinical decision-making; (3) phylogenetic analysis indicated possible clonal transmission of serotype 2 and 7 strains within China, highlighting an ongoing epidemiological trend; (4) our data provide an updated and detailed overview of serotype distribution and antimicrobial resistance patterns, particularly focusing on Shandong Province during 2023–2024, complementing the existing national surveillance landscape.

## 2. Materials and Methods

### 2.1. Isolation and Identification of R. anatipestifer

The samples used in this study were provided by Jinan Qilu Animal Health Company (Jinan, China). The samples were transported with ice packs and delivered to the laboratory for bacterial isolation, purification and identification.

In this study, brain tissues of ducks and chicken embryos were collected from Shandong, Jiangsu, Guangdong, Henan, Hebei and Anhui provinces from 2023 to 2024. Among these, Shandong Province served as the primary sampling region for this study. A purposive sampling strategy was employed, with specimens collected from multiple major poultry-farming cities across the province to reflect the regional diversity of bacterial strains. All isolates successfully obtained from clinical cases were included in the analysis. Each tissue was preserved individually in a sterile 50 mL EP tube. The samples were surface sterilized, and a small amount of tissue homogenate was inoculated onto Columbia blood agar plates (Qingdao Haibo Biotechnology Co., Ltd., Qingdao, China) using a disposable inoculation loop under aseptic conditions. The inoculated agar plates were incubated at 37 °C with 5% CO_2_ for 24 h. Next, purification culture of the isolated single colonies was performed on tryptic soy agar (TSA) plates (10% calf serum) (Qingdao Haibo Biotechnology Co., Ltd., Qingdao, China) [4]. 16S rRNA gene-specific primers were used to perform PCR identification on *R. anatipestifer* strains after three rounds of purification. The bacterial strains used in this study are listed in Table 1.

### 2.2. Serotyping of R. anatipestifer Isolates

An appropriate number of colonies was collected from the blood agar plate using an inoculation loop and resuspended in a 2 mL sterile EP tube containing 300 μL of sterile normal saline (the final concentration of the bacterial solution was 1 × 10^10^ CFU/mL to 1 × 10^12^ CFU/mL). A volume of 3 μL of the bacterial suspension was added onto a clean glass slide with an area of 3 to 5 mm^2^, followed by mixing with an equal volume of standard positive serum. The agglutination reaction was observed, and the results were interpreted within 1 min.

### 2.3. Antimicrobial Susceptibility Testing (AST) of R. anatipestifer

We determined the minimum inhibitory concentrations (MICs) of antimicrobial agents against 88 *R. anatipestifer* strains via the broth microdilution method. Given the absence of species-specific Clinical and Laboratory Standards Institute (CLSI) breakpoints for *R. anatipestifer*, MICs were interpreted using established breakpoints for related bacterial pathogens, as detailed in Table 2. A total of 14 antimicrobial agents were selected in this study, including tetracyclines (doxycycline, tetracycline), aminoglycosides (amikacin, kanamycin, gentamicin), quinolones (enrofloxacin, ciprofloxacin), amphenicols (florfenicol, chloramphenicol), β-lactams (cefotaxime, ceftiofur, ceftazidime), polymyxin B and rifampicin. All antimicrobial agents were initially diluted to a stock concentration of 5120 μg/mL. Briefly, *R. anatipestifer* strains were resuscitated, and single colonies were picked and inoculated into 6 mL of tryptic soy broth (TSB) medium (Qingdao Hope Bio-Technology Co., Ltd., Qingdao, China) containing 5% calf serum. The bacterial cultures were incubated at 37 °C with 220 rpm for 24 h until the OD_600_ value reached 1.0. A total of 80 μL of TSB medium (5% calf serum) was added to the first column of a 96-well microtiter plate, and 50 μL of the same medium was added to each of the remaining wells. Subsequently, 20 μL of each antimicrobial stock solution was added to the first column, followed by a two-fold serial dilution to obtain a gradient concentration range of 512 to 1 μg/mL [9]. After dilution, 50 μL of the prepared bacterial suspension was inoculated into each well. Escherichia coli ATCC 25922 was used as the quality control strain for the antimicrobial susceptibility test. The 96-well microtiter plates were incubated at 37 °C with 5% CO_2_ for 24 h. All experiments were performed in duplicate.

### 2.4. Analysis of Antibiotic Resistance Genes and Virulence Genes in R. anatipestifer

The optical density of the *R. anatipestifer* bacteria suspension was adjusted to an OD_600_ value of 1.0, and then genomic DNA was extracted using the water bath-boiling method. A volume of 500 μL of the bacterial suspension was pipetted into a 2 mL centrifuge tube, and the bacterial suspension was boiled in a water bath at 100 °C for 10 min. The boiled bacterial suspension was cooled at room temperature for 1 min, followed by centrifugation at 12,200 r/min for 1 min [12]. The supernatant was collected as the DNA template for subsequent experiments after centrifugation. The PCR amplification was performed in a 25 μL reaction system, which contained 12 μL of 2 × Master Mix, 1 μL of each forward and reverse primer, 1 μL of the extracted genomic DNA template, and sterile ddH_2_O was added to bring the total volume to 25 μL. The PCR amplification procedure was as follows: initial pre-denaturation at 94 °C for 30 s, followed by 32 cycles of denaturation at 98 °C for 10 s, primer-specific annealing for 30 s, and extension at 72 °C for 45 s. A final extension step was conducted at 72 °C for 2 min, and the amplified products were held at 16 °C for preservation [13]. Specific primers targeting the antibiotic resistance genes, including *armA* [14], *tet(X)* [15], *floR* [16], *ermF* [17], *rmtB* [18], *rmtC* [14], *aac*(*6′*)*-Ib* [19], *blaTEM-1* [20], *blaSHV-1* [21], *blaCTX-M* [22], *qnrS* [23], *qnrA* [23], *qnrB* [24], *qnrC* [23], *qnrD* [23], as well as the virulence genes, including *ompA* [25], *camp* [25], *wza* [25], *AS87_04050* [25], *Fur* [25], *SIP* [25], *TbdR*1 [25] and *luxE* [25], were used for PCR amplification of the above-mentioned genes, respectively. The primer sequences, annealing temperatures, expected amplicon sizes, and control information for each target gene are summarized in Supplementary Table S1.

### 2.5. Determination of the Median Lethal Dose of R. anatipestifer

Three-day-old Cherry Valley ducklings were reared in isolation until 7 days of age. Sixteen ducklings (eight males and eight females) were selected. Using a stratified randomization procedure with sex as the stratification factor, ducklings were randomly allocated into four experimental groups, resulting in a final composition of two males and two females per group. Each duckling was intraperitoneally injected with 0.5 mL of *R. anatipestifer* RA12 bacterial suspension at different concentrations: the bacterial inoculum was prepared from fresh cultures, and the challenge dose was verified by plate counting (CFU/mL). The challenge groups were administered with bacterial suspensions at concentrations of 10^8^ CFU/mL, 10^7^ CFU/mL, and 10^6^ CFU/mL, respectively, whereas the control group was injected with an equal volume of sterile TSB medium.

Another 16 seven-day-old ducklings were subjected to the identical grouping and inoculation protocol, and intraperitoneally injected with *R. anatipestifer* RA26 bacterial suspension. The clinical symptoms and mortality of the ducklings were monitored and recorded continuously for 7 days post-inoculation. Natural death was recorded as the endpoint; no intervention or euthanasia was performed on moribund animals. The median lethal dose (LD_50_) of each bacterial strain was calculated by using the modified Karber’s method. The sample size (*n* = 4 per dose group) was chosen based on the well-established use of Karber’s method in preliminary virulence assessments, which is designed to provide a robust estimate of LD_50_ with minimal animal use, in accordance with the “3Rs”.

### 2.6. Phylogenetic Tree Construction of Sequenced Strains

To investigate the genomic epidemiology of the isolates, two representative strains (RA16 and RA26) were selected for whole-genome sequencing (WGS), which was performed on an Illumina platform. Raw sequencing reads were quality-controlled using FastQC v0.11.9and trimmed with Trimmomatic v0.39. De novo genome assembly was conducted using SPAdes v3.15.5, and assembly quality was assessed with QUAST v5.2.0, showing >100× coverage and contig N50 > 200 kbp. For phylogenetic analysis, a dataset was constructed comprising the two newly sequenced genomes from this study and 69 publicly available *R. anatipestifer* genomes downloaded from the NCBI database. The core genome was identified by aligning all genomes using Roary v3.13.0. A maximum-likelihood phylogenetic tree was then constructed from the concatenated core gene alignment using IQ-TREE v2.2.2.1 and visualized with the Interactive Tree of Life (iTOL) web service. Using snp-dists 0.8.2, whole-genome SNP distance analysis was performed on 15 samples, including 13 NCBI-registered reference genomes (Accession Nos.: GCA_001670625.2, etc., covering strains from China, Germany, the UK, etc.), two isolated strains (RA16/RA26) in this study, and one standard reference sequence. The WGS scope covered the core genome to quantify the genetic relationships among samples.

### 2.7. Virulence Protection Test of R. anatipestifer

Based on the results of the antibiotic susceptibility test, one florfenicol-resistant strain and one florfenicol-sensitive strain of *R. anatipestifer* were selected. Bacterial suspensions of the two strains were prepared for challenging three-week-old ducklings, respectively. The experiment was divided into three groups with eight ducklings each: the first group was intramuscularly treated with florfenicol injection after bacterial challenge, the second group was only challenged (without treatment), and the third group was a blank control group. For the treatment groups, florfenicol was administered via intramuscular injection at a dose of 30 mg/kg (equivalent to 0.2 mL/kg of the commercial florfenicol injection), once daily, with two doses given at a 48 h interval. The feed intake, water consumption, body weight changes and mortality of ducklings were recorded throughout one week post-challenge.

## 3. Results

### 3.1. The Isolation Results of R. anatipestifer

**Figure 1:**
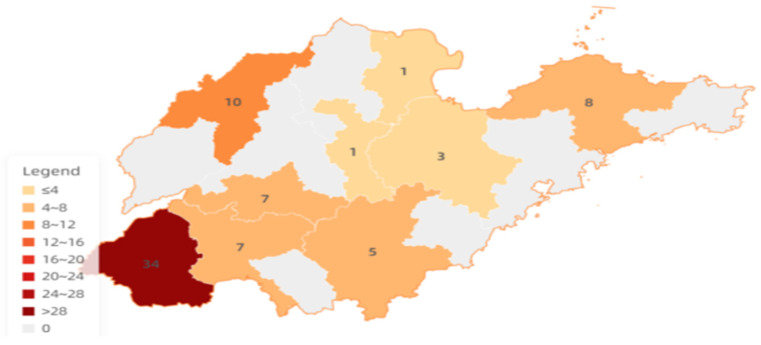
Distribution of strain counts and serotypes isolated from different regions of Shandong Province from 2023 to 2024.

A total of 88 *R. anatipestifer* strains were collected in this study, of which 50 strains were isolated in 2024, and 38 strains were isolated in 2023. Among these strains, 80 were duck-derived isolates, and 8 were of chicken-derived isolates. All duck-derived strains were isolated from duck heads. For the chicken-derived strains, four strains were isolated from the oviducts, and another four strains were isolated from chicken embryos (Table 3). In terms of geographical distribution, 76 strains were isolated from Shandong Province, including Heze, Dezhou, Yantai, Taian, Jining, Linyi, Weifang, Zibo and Dongying cities. The remaining 12 strains (13.64%) were isolated from other provinces (Figure 1).

### 3.2. Serotyping of R. anatipestifer Isolates

The serotypes of *R. anatipestifer* in China were mainly 1, 2, 5, 6, 7 and 10. In this study, the serotypes of 88 strains of *R. anatipestifer* were detected, of which 31 (35.2%) were serotype 5, 17 (19.3%) were serotype 1, 15 (17.0%) were serotype 7, 12 (13.6%) were serotype 2, 3 (3.4%) were serotype 6, and 2 (2.3%) were serotype 10 (Table 4). The main serotypes in the Shandong area are type 1 and type 5. Heze had the largest variety of serotypes, with five serotypes, including types 5, 1, 2, 7 and 10. This was followed by Jining, which included types 5, 7, 2 and 6. Dezhou was dominated by serotype 7, with serotypes 2 and 5 also isolated. Taian included types 5, 1 and 2. All strains in Yantai were serotype 1, only serotype 5 was detected in Weifang and Dongying, and one strain of serotype 7 was isolated in Zibo (Table 5).

### 3.3. Results of Antimicrobial Susceptibility Test

According to the antimicrobial susceptibility test results, all *R. anatipestifer* serotypes exhibited high resistance to amikacin, kanamycin, and polymyxin B. The gentamicin resistance rates of type 1 and type 2 strains were extremely high, reaching 82.4% and 83.3% respectively. Type 1, type 2, and type 7 strains demonstrated severe resistance to tetracycline-class drugs: the resistance rates of type 1 strains to tetracycline and doxycycline were 88.2% and 94.1%, respectively; for type 2 strains, the rates were 91.7% (tetracycline) and 83.3% (doxycycline), and type 7 strains showed an 86.7% resistance rate to both agents. All serotypes displayed extremely high resistance to quinolones, with the resistance rate of most strains exceeding 90%. Specifically, type 2 strains had a 100% resistance rate to enrofloxacin, while type 6 and type 10 strains showed 100% resistance to ciprofloxacin. Type 1 strains exhibited relatively high resistance to cefotaxime (76.5%), ceftiofur (70.6%), and ceftazidime (64.7%). In contrast, type 5 strains had a lower cefotaxime resistance rate (41.9%), and type 2 strains showed a 25% resistance rate to ceftazidime. Additionally, strains of other serotypes had resistance rates above 50% to β-lactam antibiotics. *R. anatipestifer* strains showed relatively high sensitivity to rifampicin: the resistance rates of type 1, type 2, type 5, and type 7 strains were 23.5%, 8.3%, 12.9% and 6.7%, respectively, while type 6 and type 10 strains were fully susceptible to rifampicin.

**Figure 2:**
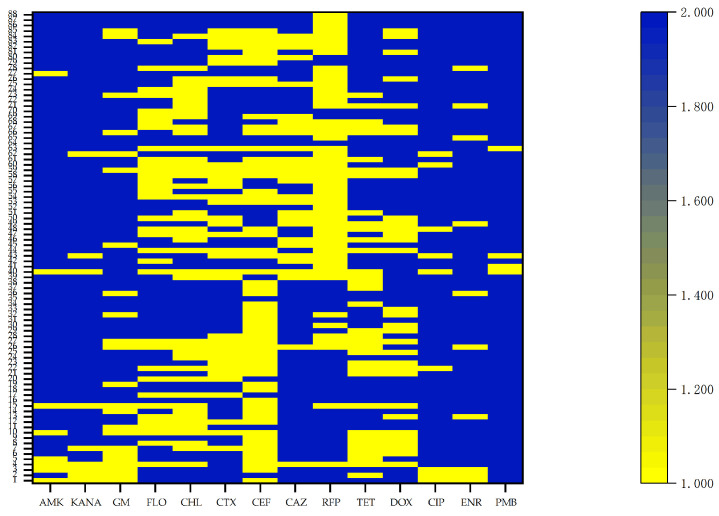
Heat map of the resistance of 88 strains isolated from 2023 to 2024 to 14 kinds of antibiotics. Indicator 1 represents sensitivity, and Indicator 2 represents drug resistance. AMK: amikacin; KANA: kanamycin; GM: gentamicin; FLO: florfenicol; CHL: chloramphenicol; CTX: cefotaxime; CEF: ceftiofur; CAZ: ceftazidime; RFP: rifampicin; TET: tetracycline; DOX: doxycycline; CIP: ciprofloxacin; ENR: enrofloxacin; PMB: polymyxin B.

The results showed that *R. anatipestifer* exhibited the highest resistance rate to polymyxin B (97%), followed by amikacin (91%), kanamycin (90%), gentamicin (74%), ciprofloxacin (90%), and enrofloxacin (89%). The resistance rates to tetracycline and doxycycline were 80% and 75%, respectively, while those to chloramphenicol and florfenicol were 47% and 55%, respectively. More than half of the strains exhibited high resistance to β-lactam drugs, with resistance rates of 60% to cefotaxime, 53% to ceftiofur, and 55% to ceftazidime. *R. anatipestifer* strains showed high susceptibility to rifampicin, with a low resistance rate of 22%. Additionally, the resistance of *R. anatipestifer* to aminoglycoside drugs was more severe in 2024 compared with 2023 (Figure 2).

**Figure 3:**
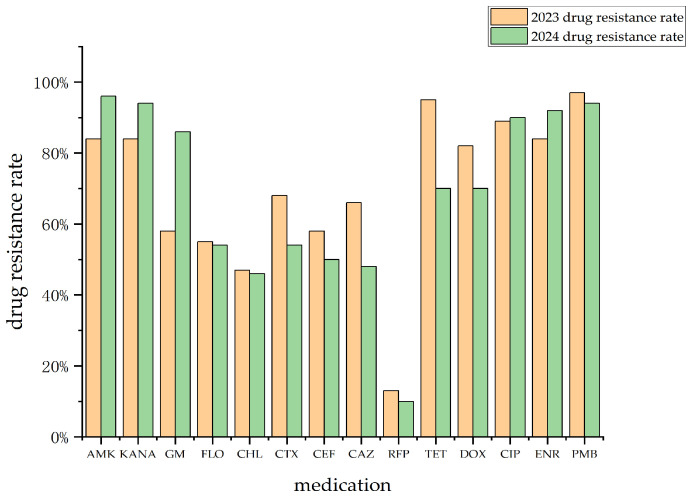
Comparison of drug resistance rates of strains isolated in 2024 and 2023 against 14 different antimicrobial agents. AMK: amikacin; KANA: kanamycin; GM: gentamicin; FLO: florfenicol; CHL: chloramphenicol; CTX: cefotaxime; CEF: ceftiofur; CAZ: ceftazidime; RFP: rifampicin; TET: tetracycline; DOX: doxycycline; CIP: ciprofloxacin; ENR: enrofloxacin; PMB: polymyxin B.

The Wilcoxon signed-rank test (*α* = 0.05) revealed no statistically significant difference in the overall antimicrobial resistance rates of *R. anatipestifer* to 14 tested antibacterial agents between 2023 and 2024 (*Z* = −1.028, *p* = 0.304 > 0.05). Notably, resistance to aminoglycosides (AMK, KANA, GM) increased significantly from 84%, 84%, and 58% in 2023 to 96%, 94%, and 86% in 2024, respectively. In contrast, considerable reductions were detected in TET (from 95% to 70%), DOX (from 82% to 70%), and cephalosporins (CTX: from 68% to 54%; CEF: from 58% to 50%; CAZ: from 66% to 48%). Resistance to FLO, CAP, CIP, and ENR remained stable, with fluctuations not exceeding 2% (Figure 3).

**Figure 4:**
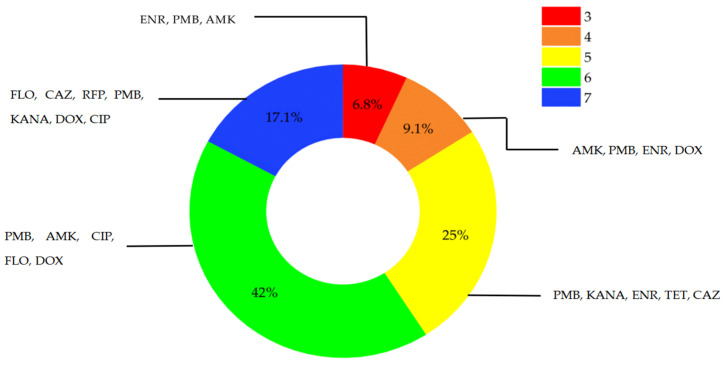
Proportion of multidrug-resistant strains and their main resistance types. AMK: amikacin; KANA: kanamycin; FLO: florfenicol; CAZ: ceftazidime; RFP: rifampicin; TET: tetracycline; DOX: doxycycline; CIP: ciprofloxacin; ENR: enrofloxacin; PMB: polymyxin B.

All strains in this study were multidrug-resistant (MDR) strains. Among them, 4 strains exhibited resistance to three antimicrobial agents, 14 strains to four agents, and 24 strains to five agents. Resistance to six antimicrobials was the most prevalent phenotype (41 strains), while five strains showed resistance to seven antimicrobials (PMB, AMK, and CIP). Resistance to five antimicrobials was mainly linked to PMB, KANA and ENR. Additionally, multidrug resistance to seven antimicrobials was predominantly associated with FLO, CAZ, RFP, PMB, KANA, DOX and CIP (Figure 4).

### 3.4. Analysis of Antibiotic Resistance Genes and Virulence Genes of R. anatipestifer

**Figure 5:**
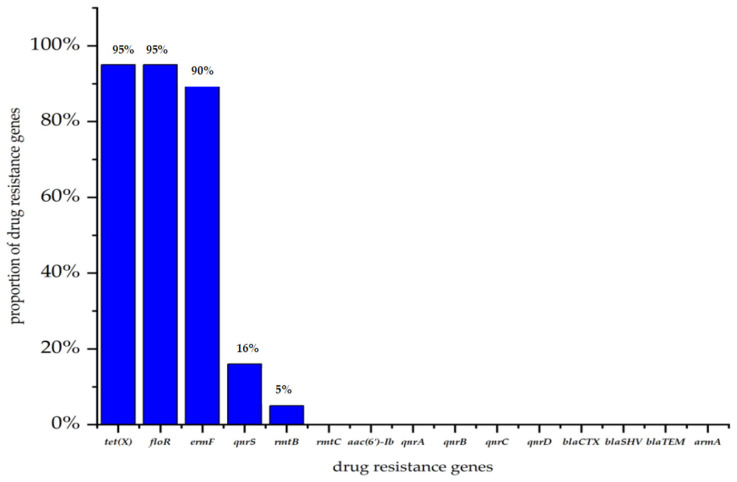
Detection rates of 15 resistance genes in 88 strains.

**Figure 6:**
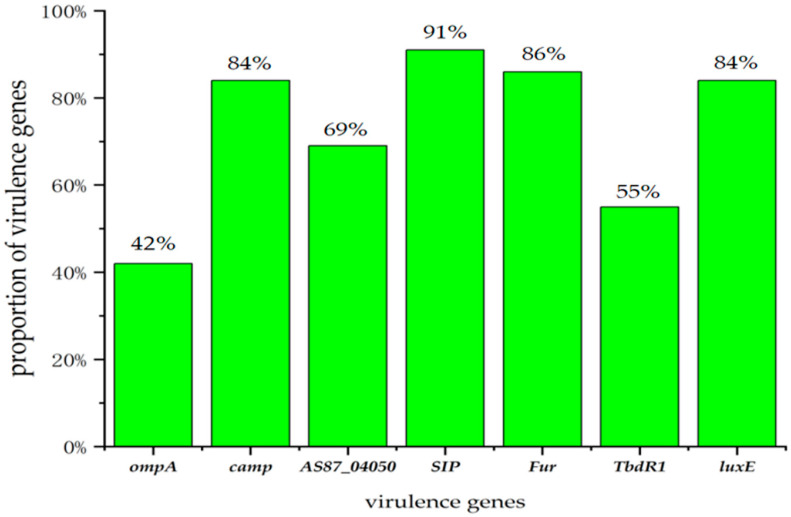
Results of the detection of 8 virulence genes in 88 isolated obtained from 2023 to 2024.

Among the 88 *R. anatipestifer* strains, a total of five resistance genes and seven virulence genes were detected (Figure 5 and Figure 6). For the identified resistance genes, *tet(X)* and *floR* exhibited the highest detection rates (*n* = 84 for both), followed by *ermF* (*n* = 79), *qnrS* (*n* = 14) and *rmtB* (*n* = 4). No other resistance genes were identified in the tested strains. Among the virulence genes, *SIP* had the highest detection rate (*n* = 80), followed by *Fur* (*n* = 76), *camp* and *luxE* (*n* = 74 for both), *AS87*_04050 (*n* = 61), *TbdR1* (*n* = 48), and *ompA* (*n* = 37). The virulence gene *wza* was not detected in any of the strains.

### 3.5. Correlation Analysis Between Resistance Genotypes and Phenotypes

A notable discordance was observed between the presence of the *tet(X)* gene and phenotypic resistance to tetracyclines. Among the 84 *tet(X)*-positive isolates, only 65 (77.4%) exhibited resistance to tetracycline, and 70 (83.3%) to doxycycline, indicating that 19 (22.6%) and 14 (16.7%) of *tet(X)*-positive strains, respectively, remained phenotypically susceptible.

Statistical analysis using Fisher’s exact test revealed no significant correlation between the presence of the *tet(X)* gene and resistance to tetracycline (*p* = 0.172) or doxycycline (*p* = 0.106). Similarly, the carriage of *floR* was not significantly associated with florfenicol resistance (*p* = 0.754). However, a strong and significant correlation was found between the presence of *ermF* and resistance to both tested macrolide–lincosamide-related drugs (if tested) (*p* < 0.001).

Furthermore, we explored the potential link between multidrug resistance (MDR) profiles and the number of virulence genes carried. Isolates exhibiting resistance to a broader spectrum of antibiotics (e.g., ≥6 drug classes) tended to carry a higher median number of virulence genes (median = 5, IQR: 4–6) compared to those with a narrower resistance profile (≤3 drug classes; median = 3, IQR: 2–4). The Kruskal–Wallis test indicated this difference was statistically significant (*p* < 0.01).

### 3.6. The Determination Result of the Median Lethal Dose of R. anatipestifer

The challenge test indicated that the LD_50_ of strain RA26 (carrying *ompA*, *camp*, *SIP*, *Fur*, and *luxE*) was 2.57 × 10^7^ CFU/mL, while that of strain RA12 (carrying *camp*, *SIP*, and *luxE*) was 2.75 × 10^7^ CFU/mL (Table 6). No statistically significant difference in clinical virulence was observed between these two strains.

### 3.7. Genomic Characteristics of Riemerella anatipestifer Isolates

**Figure 7:**
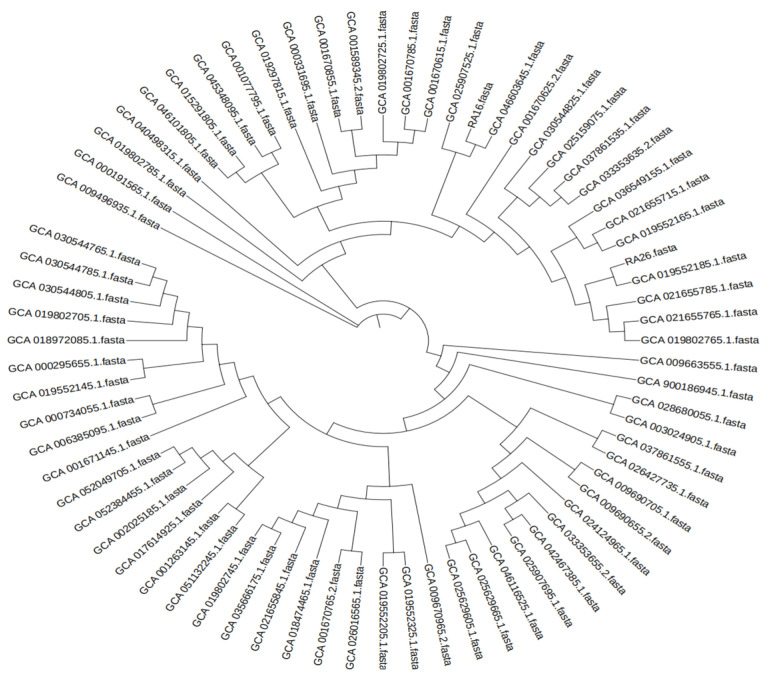
The phylogenetic tree constructed with 71 strains.

Phylogenetic tree analysis revealed that all strains were clustered into two distinct clades with different serotypes: RA26, which belonged to serotype 7 and was closely related to 20190403E1-1 (a goose-derived isolate from Jiangsu Province, China); RA16, which belonged to serotype 2 and was closely related to JW1 (from Guangdong Province, China), and ZWRA168 (a goose-derived strain from Jiangsu Province, China). The serotypes 2 and 7 identified in this study have exhibited clonal transmission in China. These two strains exhibited a large phylogenetic divergence from the strains isolated in Germany and the United Kingdom (Figure 7). The SNP distances among strains ranged from 0 to 23,751, and all strains were divided into two independent clades: the closely related clade contained 14 strains (predominantly domestic strains, including RA16/RA26), with intra-clade SNP distances ≤92, indicating high genetic homology; the distant clade only included the United Kingdom strain GCA_017614925.1, with SNP distances >23,600 from the closely related clade, suggesting a distant evolutionary relationship. Within the closely related clade, the SNP distance between GCA_019802765.1 and GCA_021655765.1 was 0, and that between GCA_019552185.1 and GCA_021655785.1 was only 9. The SNP distances of RA16 with domestic strain GCA_046603645.1 and RA26 with GCA_019552185.1 were 16 and 19, respectively. Both RA16 and RA26 showed close clustering with domestic strains but significant divergence from other countries strains. The SNP distances between the reference sequence and domestic strains ranged from 78 to 92, confirming its applicability as a standard reference.

### 3.8. Virulence Protection Assay in Ducklings

The ducklings in this experiment presented clinical symptoms of depression, lacrimation, coughing, and eyelid swelling at 12 h post-challenge. The dead ducklings were observed to have typical pathological lesions, including pericarditis, hepatitis, meningitis, and fibrinous airsacculitis. Within one week post-challenge, the challenged ducklings showed a significant reduction in feed and water intake, accompanied by retarded body weight gain. Mortality was only observed in the challenged groups within 120 h post-challenge. Specifically, the ducklings challenged with the florfenicol-resistant strain showed a mortality rate of 87.5% following florfenicol treatment, while those challenged with the susceptible strain had a mortality rate of 37.5% after the same treatment (Table 7). These findings demonstrated that the in vivo therapeutic efficacy of florfenicol was consistent with the results of in vitro drug susceptibility testing against it. Furthermore, no *R. anatipestifer* was isolated in the brain, liver, spleen, and fecal samples collected from the recovered ducklings after treatment.

## 4. Discussion

*R. anatipestifer* is a Gram-negative bacterium belonging to the genus *Flavobacterium*, which is predominantly transmitted among waterfowl and predominantly infects ducklings under 8 weeks of age. In recent years, however, *R. anatipestifer* has been identified transmitting directly among avian species, including chickens and geese, as well [26]. This study corroborates this perspective by isolating eight strains of *R. anatipestifer* from chicken oviducts and embryos. Meanwhile, the pathogen is primarily transmitted horizontally via the respiratory tract and wounds. Reports on the vertical transmission of *R. anatipestifer* are scarce; however, its detection in oviducts and embryos in this study suggests a potential risk of egg-borne transmission. Furthermore, genomic studies have revealed a high degree of genetic similarity between duck- and goose-derived strains, indicating their potential for cross-species transmission among poultry. Consequently, these findings suggest that control strategies must extend beyond waterfowl, incorporating the monitoring of breeder chicken flocks and egg disinfection into comprehensive prevention and control systems. This pathogen exhibits a large number of serotypes with no cross-protection among different serotypes, and the prevalent serotypes vary across different regions, which further increases the difficulty of vaccine-based prevention and control against *R. anatipestifer* infection. In addition, the antimicrobial resistance of *R. anatipestifer* has been escalating year by year due to the irrational overuse of antibiotics in poultry production. Therefore, investigations into the resistance genes are of great significance for controlling the spread and variation of antimicrobial-resistant strains in poultry flocks.

According to the survey results, among the 376 strains isolated by Wei et al. [12], the resistance rate of RA to β-lactams was 27.7% (104/376). The resistance rate of strains isolated from 2010 to 2015 was 12.9%, while that of strains isolated from 2016 to 2020 was 29.0%. The resistance rate of the strains isolated in this study to β-lactam was 58%, which was twice as high as the results reported by Wei Xinyi and others. In 2023, Liu et al. [4] reported that more than 50% of the 74 collected *R. anatipestifer* strains exhibited resistance to cefotaxime. In this study, the resistance rate to cefotaxime was 61%. These findings further confirm that the antimicrobial resistance of *R. anatipestifer* to β-lactams has been increasing continuously in recent years.

In 2022, Zhang Chunxiao et al. isolated 70 goose-derived strains in Hebei. The resistance rates of these strains to kanamycin and gentamicin were 100%, and the resistance rate to amikacin was 91.1% [8]. The test results of the strains collected by Yang Zhishuang et al. between 1994 and 2021 showed that over 90% of them were resistant to kanamycin, gentamicin, and quinolones, and the resistance rate to tetracycline was 88% [7]. Among the 171 strains collected by Zero Lyu et al. in Shandong from 2022 to 2023, the resistance rates to gentamicin, enrofloxacin and kanamycin were 77%, 73%, and 62% respectively [4]. All these were multidrug-resistant strains, with the largest number of strains being resistant to five antibiotics. They all demonstrated a high degree of drug resistance, which is similar to the high resistance results of this study.

This escalating trend is closely linked to antimicrobial use practices in China’s livestock sector. According to relevant reports, β-lactams and aminoglycosides have been commonly used for preventing and treating bacterial infections in poultry, including waterfowl [27,28]. The sustained selection pressure from such use, including non-prudent practices, is a recognized driver for antimicrobial resistance (AMR) development [29,30]. The high prevalence of resistance genes *tet(X)*, *floR*, and *ermF* in our isolates (95.5%, 95.5%, and 89.8%, respectively) provides molecular evidence of this pressure, contributing to the high multidrug resistance (MDR) rate observed.

The diminishing efficacy of conventional drugs leads to significant clinical and economic implications. Increased resistance elevates the risk of treatment failure and mortality, necessitating the use of more costly or last-resort alternatives [31]. This directly translates to reduced productivity and heightened economic losses in poultry farming [31]. Consequently, guiding antimicrobial therapy through localized susceptibility surveillance, as emphasized by this study, is crucial for mitigating AMR and safeguarding both animal health and economic sustainability.

Reports on the quinolone resistance mechanism of *R. anatipestifer* remain relatively scarce, leaving substantial scope for further investigation. Quinolone resistance can be mediated by multiple mechanisms, including the overexpression of multidrug resistance (MDR) efflux pumps, reduced membrane permeability, and the acquisition of plasmid-mediated quinolone resistance (PMQR) genes [32]. Additionally, non-quinolone-resistance-determining region (non-QRDR) mutations may also exert a notable impact on the function of [33,34]. Epidemiological data indicate that resistance rates of *R. anatipestifer* to ciprofloxacin and enrofloxacin reached 97.8% and 99.3%, respectively, during 2013–2018 [35], highlighting the severity of quinolone resistance in this pathogen. In the present study, five pairs of specific primers targeting the PMQR genes *qnrA*, *qnrB*, *qnrC*, and *qnrD* were designed and used for detection; however, no positive amplifications were observed for these genes. The strains isolated in Thailand by Pathomchai-Umporn C et al. have not been found to carry the *qnrA* or *qnrB* resistance genes [36]. The gene detection results of the strains isolated by Lyu, Z et al. also did not identify *qnrA* and *qnrB* [37]. Another study conducted in China showed that none of the 103 *R. anatipestifer* strains tested harbored quinolone resistance genes [38]. These findings suggest that PMQR mechanisms are not responsible for the quinolone resistance of the tested *R. anatipestifer* isolates, and resistance is more likely associated with point mutations in the QRDR. Consistent with previous reports, the substitution at amino acid position 83 of the GyrA protein is recognized as the primary mutation site mediating fluoroquinolone resistance in bacteria [35].

Studies have demonstrated that three categories of inactivating enzyme genes (e.g., *aac*(*6′*)*-Ib*, *aac*(*6′*)*II*, *aph*(*3′*)*IIα*), and 16S methyltransferase genes confer resistance to gentamicin, whereas the *aac*(*6′*)*-Ib* gene also mediates resistance to kanamycin [39]. In the present study, the resistance rate of *R. anatipestifer* isolates to kanamycin reached 91%, with that to gentamicin recorded as 72%, indicating a high-level-of-resistance gene was not detected in any of the tested isolates. These findings imply that the high-level aminoglycoside resistance observed in this study may be attributed to other uncharacterized resistance mechanisms or the presence of alternative resistance genes.

This study confirms a significant discordance between the presence of key resistance genes and the corresponding phenotypic resistance, most notably for *tet(X)*. While *tet(X)* encodes a potent tetracycline-inactivating enzyme [15], approximately 20% of *tet(X)*-positive isolates in our collection remained susceptible to tetracyclines. This finding, supported by the lack of statistical correlation (see Section 3.5), underscores that gene presence alone does not guarantee expression or a resistant phenotype, a phenomenon increasingly recognized in bacterial genomics [40]. Several mechanistic explanations could account for this: 1. Gene Silencing or Low Expression: The *tet(X)* gene may be located in a chromosomal context with a weak promoter, or its expression could be suppressed by regulatory elements such as repressor proteins. Epigenetic silencing has also been reported for some resistance genes in other pathogens [41]. 2. Genetic Context and Mobile Elements: The gene might be located on a plasmid or genomic island that is not stably maintained or is poorly expressed in *R. anatipestifer*. The absence of necessary transcriptional activators or the presence of genetic interruptions (e.g., insertion sequences) could render the gene non-functional, as seen in studies of other antibiotic resistance genes [42]. 3. Physiological Fitness Cost: High-level expression of resistance determinants like *tet(X)* can impose a metabolic burden on the bacterium. In the absence of antibiotic selection pressure, strains may downregulate or inactivate such genes to maintain fitness, leading to a susceptible phenotype despite gene carriage, a well-documented evolutionary trade-off [43]. In stark contrast, the presence of the *ermF* gene showed a high degree of concordance with the resistant phenotype. This suggests that in these isolates, *ermF* is likely constitutively expressed—meaning its resistance mechanism is active regardless of antibiotic selection pressure. This stable expression pattern is probably due to its favorable genetic context, such as integration into the chromosome or a stable plasmid, consistent with previous reports identifying *ermF* as a reliable resistance marker in *R. anatipestifer* [17].

While resistance gene expression is regulated by the above factors, virulence-associated genes also play a role in the biological characteristics of *R. anatipestifer.* Hu et al. constructed an *ompA*-deleted mutant of *R. anatipestifer* Th4 and confirmed that *ompA* deletion reduced the adhesion ability of the mutant to Vero cells and attenuated its pathogenicity in ducklings [44]. Wang et al. established a transposon (Th4351) insertion mutant library of *R. anatipestifer* Yb2, and sequencing identified 49 genes with insertion inactivation. Insertional inactivation of the *AS87_04050* gene resulted in the loss of serum agglutination ability of the strain, confirming that this gene is involved in lipopolysaccharide synthesis and the pathogenicity of *R. anatipestifer* [45]. These findings collectively demonstrate that *ompA* is a critical virulence factor of *R. anatipestifer*. In addition, the Fur gene regulates the expression of enzymes associated with the tricarboxylic acid (TCA) cycle, aromatic amino acid synthesis, and purines and nucleotides metabolism by modulating iron uptake systems, redox reactions, and the type IX secretion system, thereby affecting the virulence of *R. anatipestifer* [46].

Our statistical analysis indicates a significant trend where isolates with broader MDR profiles carry a greater number of virulence genes (see Section 3.5). This suggests a potential co-selection or genetic linkage between resistance and virulence determinants in *R. anatipestifer*. This alignment could be driven by: 1. Genetic Linkage: Resistance genes and virulence genes may be located on the same mobile genetic elements (e.g., plasmids, genomic islands). Selection pressure from antibiotic use not only enriches for resistance but also concurrently promotes the spread of linked virulence factors, a mechanism reported in major pathogens like *E. coli* and *Salmonella* [47]. 2. Global Regulatory Networks: Stress responses triggered by antibiotics or host environments may upregulate both resistance mechanisms (like efflux pumps) and virulence factor expression through shared regulatory pathways (e.g., two-component systems, MarA, SoxS) [48]. 3. Fitness Advantage in Hostile Environments: Strains accumulating both resistance and virulence traits may have a selective advantage in clinical settings under dual pressures from antibiotic treatment and host immune defenses, leading to the emergence of “high-risk” clones [49]. This convergence poses a serious public health concern, as it implies that aggressive antibiotic use in poultry might inadvertently promote the evolution of *R. anatipestifer* strains that are not only harder to treat but also potentially more pathogenic, mirroring trends observed in other bacterial species [49].

Due to the absence of internationally standardized breakpoints for *R. anatipestifer*, all current studies on its antimicrobial resistance refer to breakpoints established for other bacterial strains. As a result, antimicrobial susceptibility testing for *R. anatipestifer* has certain limitations. While it can indicate relatively high resistance or sensitivity trends, the findings cannot be used as definitive references for clinical medication. The selection of florfenicol was based on both the results of susceptibility testing and the drugs commonly used in current clinical practice. Therefore, investigating the consistency between in vitro efficacy and in vivo susceptibility testing for florfenicol is particularly important, as it would enhance the accuracy of antimicrobial susceptibility testing. In the present study, no significant differences were observed in the results of the *R. anatipestifer* animal challenge test; however, the mortality rate of the challenged group showed no significant differences; however, during the treatment trial, the mortality rate reached 100% during the subsequent treatment trial. On the one hand, according to relevant reports, the incidence of *R. anatipestifer* is influenced by temperature and humidity, with higher rates observed in winter and spring [50,51]. However, systematic data on this epidemiological pattern remain relatively scarce. Therefore, we hypothesize that the discrepancy in duckling mortality outcomes in the present study may be associated with seasonal variations. On the other hand, the high mortality rate (87.5%) observed in ducklings challenged with the florfenicol-resistant strain (RA7) and treated with florfenicol can be directly attributed to the strain’s resistant phenotype, as confirmed by its elevated MIC value. The standard florfenicol regimen administered, while therapeutically effective against the susceptible strain (RA25), was insufficient to overcome the high-level resistance conferred by mechanisms such as the *floR* gene carried by RA7. Collectively, these findings provide valuable insights into identifying the optimal timing for antibiotic-based prevention and control strategies against *R. anatipestifer* infections.

Based on core-genome phylogenetic analysis of 71 strains (including two from this study), clonal transmission of specific serotypes in China was confirmed. Notably, serotype 2 strain RA16 from Shandong clustered with strains JW1 (Guangdong) and ZWRA168 (Jiangsu) with 100% bootstrap support, while serotype 7 strain RA26 from Shandong formed a monophyletic clade with strain 20190403E1-1 (Jiangsu). This interprovincial clustering indicates clonal expansion and regional dissemination, likely associated with the movement of live birds and poultry products. These findings have several important implications for surveillance and control: First, cross-provincial surveillance is needed to track these dominant clones (serotypes 2 and 7). Second, despite minimal cross-protection between serotypes, the high genetic homogeneity within each clone suggests that vaccines targeting their conserved genomic regions could be effective against strains of the same lineage. Third, the clear phylogenetic separation of Chinese clones from European and American strains underscores that control strategies must be informed by local genomic epidemiology.

Limitations of the study: This study has several limitations that should be considered when interpreting the results. First, the absence of species-specific clinical breakpoints for *R. anatipestifer* necessitated the adoption of criteria from related pathogens (*P. multocida*, *E. coli*). Consequently, the classifications of “resistant” and “susceptible” herein should be viewed as phenotypic profiles based on selected microbiological criteria rather than definitive predictors of clinical outcome. Second, the determination of LD_50_ values, while providing essential comparative virulence data for the tested strains, was based on a limited number of animals per dose group (*n* = 4). This sample size, chosen in adherence to the ‘Reduction‘ principle of the 3Rs and consistent with preliminary assessments in avian pathogen studies, yields a reliable estimate for within-study comparison but may affect the precision of the LD_50_ point estimate compared to larger-scale standardized protocols. Third, although sexes were equally represented in the challenge design (two males and two females per group) to account for basic biological variability, the very small subgroup size (*n* = 2 per sex per dose) precluded any meaningful statistical analysis of sex as a biological variable. No consistent, overt sex bias in mortality was observed during the trial, but this does not rule out more subtle sex-related effects that could be elucidated in future studies with dedicated, sex-balanced larger cohorts. Finally, the epidemiological data are primarily derived from Shandong Province over two years; thus, the findings may not fully represent the national prevalence or long-term trends of *R. anatipestifer*. Future studies incorporating pharmacokinetic/pharmacodynamic data to establish clinical breakpoints, larger animal cohorts for precise virulence modeling, and expanded spatiotemporal surveillance will be crucial to address these limitations and further guide antimicrobial stewardship and disease control.

## 5. Conclusions

A total of 88 strains were isolated in this study, including serotypes 1, 2, 5, 6, 7, and 10. Serotypes 1 and 5 were predominantly distributed in Shandong Province. The results of antimicrobial susceptibility testing (AST) against 14 antimicrobial agents showed that the strains were relatively sensitive to rifampicin, whereas the resistance rates to the remaining antibiotic agents all exceeded 40%. Five resistance genes (*tet(X)*, *floR*, *ermF*, *qnrS*, *rmtB*) and seven virulence genes (*ompA*, *camp*, *AS87_04050*, *SIP*, *Fur*, *TbdR*1, *luxE*) were found in total. Phylogenetic tree analysis showed that RA26 and strain 20190403E1-1, as well as RA16 and JW1, each had a bootstrap value of 100% and clustered within the same phylogenetic group. These strains exhibited a significant phylogenetic divergence from those isolated in the UK and Germany. The results of the challenge test showed that the LD_50_ of RA26 was 2.57 × 10^7^ CFU/mL, and that of RA12 was 2.75 × 10^7^ CFU/mL. The virulence protection test results indicated that the outcomes of in vitro antimicrobial susceptibility testing were consistent with the in vivo therapeutic efficacy, and *R. anatipestifer* was not detected in the visceral organs of surviving ducklings.

## Figures and Tables

**Table 1:** The strains information used for challenge, sequencing, and virulence protection assays in this study.

Strain Designation	Year of Isolation	Source	Serotype	Key Virulence	Whole-Genome Sequenced (WGS)	Median Lethal Dose	Virulence Protection Test
RA12	2024	Taian	5	*camp*, *SIP*, *luxE*	No	Yes	No
RA26	2024	Dezhou	7	*ompA*, *camp*, *SIP*, *Fur*, *luxE*	Yes	Yes	No
RA16	2024	Heze	2	*ompA*, *AS87_04050*, *SIP*, *Fur*, *TbdR1*, *luxE*	Yes	No	No
RA25	2024	Heze	2	*ompA*, *camp*, *AS87_04050*, *SIP*, *Fur*, *TbdR1*, *luxE*	No	No	Yes
RA7	2024	Shangqiu	2	*ompA*, *camp*, *AS87_04050*, *SIP*, *Fur*, *TbdR1*, *luxE*	No	No	Yes

**Table 2:** The resistance breakpoints for the 14 antibiotics used as reference criteria.

Antimicrobial Class	Antimicrobial Agent	Resistant (R)≥	Reference Standard
Aminoglycosides	Amikacin	64	CLSI VET01S Ed6 [10]
	Gentamicin	16	CLSI VET01S Ed6
	Kanamycin	64	CLSI VET01S Ed6
Fluoroquinolones	Enrofloxacin	1	CLSI VET01S Ed6
	Ciprofloxacin	4	CLSI M100-Ed34 [11]
Tetracyclines	Tetracycline	16	CLSI VET01S Ed6
	Doxycycline	16	CLSI VET01S Ed6
Amphenicols	Florfenicol	8	CLSI VET01S Ed6
	Chloramphenicol	32	CLSI M100-Ed34
β-Lactams	Cefotaxime	4	CLSI M100-Ed34
	Ceftiofur	8	CLSI VET01S Ed6
	Ceftazidime	16	CLSI M100-Ed34
Polymyxin	Polymyxin B	4	CLSI M100-Ed34
Ansamycins	Rifampicin	4	CLSI M100-Ed34

**Table 3:** *R. anatipestifer* isolated from different sources.

Year	Animal Origin	Visceral Source	Separation Quantity
2023	Duck	Duck head	30
	Chicken	Oviduct	4
	Chicken	Chicken embryos	4
2024	Duck	Duck head	50

**Table 4:** Serotype analysis of *R. anatipestifer*.

Serotype	The Number of Isolated Strains	Percentage
1	17	19.3%
2	12	13.6%
5	31	35.2%
6	3	3.4%
7	15	17.0%
10	2	2.3%

**Table 5:** Distribution proportion of *R. anatipestifer* serotypes among different cities of Shandong Province.

Region	Serotype/Proportion
Heze	type 5: 17%, type 1: 8%, type 2: 6%, type 7: 5%, type 10: 1%
Dezhou	type 7: 6%, type 2: 1%, type 5: 1%
Yantai	type 1: 9%
Taian	type 5: 5%, type 1: 1%, type 2: 1%
Jining	type 5: 5%, type 7: 1%, type 2:1%, type 6: 1%
Linyi	type 5: 3%, type 2: 2%
Weifang	type 5: 2%
Dongying	type 5: 1%
Zibo	type 7: 1%

**Table 6:** Results of duckling challenge test.

	CFU	0~24	24~48	48~72	72~96	96~120	120~144	144~168
RA12	5.5 × 10^8^	0	2	1	0	0	0	0
	5.5 × 10^7^	0	0	1	0	1	0	0
	5.5 × 10^6^	0	0	0	1	0	0	0
LD_50_	2.75 × 10^7^ CFU/mL							
RA26	1.61 × 10^8^	0	1	0	0	1	0	0
	1.61 × 10^7^	0	0	0	1	1	0	0
	1.61 × 10^6^	0	0	0	0	0	0	0
LD_50_	2.57 × 10^7^ CFU/mL							
Control group		0	0	0	0	0	0	0
		0	0	0	0	0	0	0
		0	0	0	0	0	0	0

**Table 7:** Daily average feed and water intake of ducklings.

	Average Food Intake (g)	Average Water Intake (mL)	Average Body Weight (g)	Number of Deaths
RA7				
treatment group	49.7	146	64.1	7/8
Challenge group	65.6	150	62.3	8/8
Control group	95.8	150	69.6	0/8
RA25				
treatment group	68.2	150	60.7	3/8
Challenge group	36.7	145	57.6	8/8
Control group	95.8	150	73.8	0/8

## Data Availability

Data is contained within the article.

## Supplementary Materials

The following supporting information can be downloaded at: https://www.mdpi.com/article/10.3390/ani16030442/s1, Table S1:The primer sequences and annealing temperatures used in this experiment; Table S2: Strain data retrieved from the NCBI database.
