# Supplementary material for: Study on Antimicrobial Resistance and Molecular Characteristics of Riemerella anatipestifer
Source: Animals (Basel). 2026 Jan 31;16(3):442. doi: 10.3390/ani16030442 (PMC12897391; doi:10.3390/ani16030442)
Supplement: Supplementary file 1 [file animals-16-00442-s001.zip › animals-4108632-supplementary.pdf]

Table S1. The primer sequences and annealing temperatures used in this experiment

| Primers | Sequences(5′-3′) | Product size/bp | Annealing temperature/℃ |
|---|---|---|---|
| *RA-16S-F* | CAGCTTAACTGTAGAACTGC | 665 | 48.6 |
| *RA-16S-R* | TCGAGATTTGCATCACTT | | |
| *arm*A-F | TTTGCGATGTGCACCAGTAA | 514 | 45.1 |
| *arm*A-R | CGATATCGTTGGTGGTGCCATA | | |
| *tet*(X)-F | ATGACAATGCGAATAGATACAGAC | 1167 | 47.7 |
| *tet*(X)-R | CAATTGCTGAAACGTAAAGTC | | |
| *floR*-F | AACCCGCCCTCTGGATCAAGTCAA | 590 | 62 |
| *floR*-R | GCACCAGCCCCAACGAAACCAGTA | | |
| *ermF*-F | AGAAATTGCCCGTTCGTTTTACGG | 460 | 55.1 |
| *ermF*-R | ATTTTTCAGCGACAACTTCCAGCATTT | | |
| *aac(6′)-Ib*-F | ATGACCTTGCGATGCTCTATGA | 486 | 53 |
| *aac(6′)-Ib*-R | CGAATGCCTGGCGTGTTT | | |
| *blaTEM*-F | AGGAAGAGTATGATTCAACA | 528 | 44.6 |
| *blaTEM*-R | CTCGTTTGGTATGGC | | |
| *qnr*S-F | ACGACATTCGTCAACTGCAA | 417 | 53.9 |
| *qnr*S-R | AACAAGCTGAAGCGCCTG | | |
| *qnr*A-F | TCAGCAAGAGGATTCTCA | 627 | 49.2 |
| *qnr*A-R | GGCAGCACTATTACTCCCA | | |
| *qnr*B-F | GATCGTGAAAGCCAGAAAGG | 469 | 53.6 |
| *qnr*B-R | ACGATGCCTGGTAGTTGTCC | | |
| *qnr*C-F | GGGTTGTACATTTATTGAATC | 447 | 53 |
| *qnr*C-R | TCCACTTTACGAGGTTCT | | |
| *qnr*D-F | CAAGATCAATTTACGGGGAAT | 582 | 52.3 |
| *qnr*D-R | AACAAGCTGAAGCGCCTG | | |
| *blaSHV*-F | GCCTTTATCGGCCTTCACTCAAG | 885 | 55.7 |
| *blaSHV*-R | TTAGCGTTGCCAGCTCGATCA | | |
| *blaCTX*-F | TTTGCGATGTGCAGTACCAGTAA | 544 | 54 |
| *blaCTX*-R | CGATATCGTTGGTGGTGCCATA | | |
| *rmtB*-F | ATGAACATCAACGATGCCC | 756 | 52.6 |
| *rmtB*-R | CCTTCTGATTGGCTTATCCA | | |
| *rmtC*-F | CGAAGAAGTAACAGCCAAAG | 711 | 51.3 |
| *rmtC*-R | ATCCCAACATCTCTCCCACT | | |
| *ompA*-F | ATGGACAAGGAGTTTATGTTG | 1164 | 46.3 |
| *ompA*-R | TTATTTTCTTTTCTTTTTTACTACT | | |
| *camp*-F | ATGAAACAATCTATTATCTTAGGTA | 1026 | 45.6 |
| *camp*-R | TTACTTTGCATTTAACTCATATC | | |
| *wza*-F | AGTCTTTAGACAAGTAGATGCC | 939 | 47.6 |
| *wza*-R | TTACTTTGCATTTAACTCATATC | | |
| *AS87*_04050-F | AGGCCGCTTTAACTCATCGTATTT | 585 | 54.3 |

| | | | |
|---|---|---|---|
| *AS87*_04050-R | CTTCGCCTATTTTCTCATCGTATTT | | |
| *Fur*-F | ATGGAACATCAAGAGAAAGATATAG | 468 | 48 |
| *Fur*-R | TTATGTTTTTTATGACCGTAGA | | |
| *SIP*-F | ATGCCTAAGACACCGAAATGGATG | 729 | 55.6 |
| *SIP*-R | TTACAAACCTTGTTTTCCTTCCAACC | | |
| *TbdR*1-F | TCGTGGGGCAATCTAAACTAAGC | 427 | 54.8 |
| *TbdR*1-R | AGCCCTGCCCAAACATCATAA | | |
| *luxE*-F | ATGCCTTCTATTTTTGATATTAACAC | 999 | 52.3 |
| *luxE*-R | CTAAGAAACCAAAAGGCTACAACC | | |

Table S 2. Strain data retrieved from the NCBI database.

| Strain name | Login ID | Collection date | Geographic location |
|---|---|---|---|
| JW1 | GCA_046603645.1 | 2018/10 | China:Guangdong |
| RCAD0125 | GCA_001670625.2 | 2012/1/14 | China:Hainan |
| RCAD0569 | GCA_030544825.1 | 2017/12/9 | China:Beijing |
| SCVM0004 | GCA_025159075.1 | 2018/10/22 | China:Chonqing |
| RCAD1083 | GCA_037861535.1 | 2020/8/25 | China:Sichuan, dujiangyan |
| RCAD1242 | GCA_033353635.2 | 2020 | China:Henan, yongcheng |
| RA-3M | GCA_036549155.1 | 2023/6/5 | China:kunming |
| 20190609E1-1 | GCA_021655715.1 | 2019/6/9 | China:Jiangsu |
| 20190109E1-1 | GCA_019552165.1 | 2019/1/9 | China:Jiangsu |
| 20190403E1-1 | GCA_019552185.1 | 2019/4/3 | China:Jiangsu |
| 20190213Y1-1 | GCA_021655785.1 | 2019/2/13 | China:Jiangsu |
| 20200501E2-1 | GCA_021655765.1 | 2020/5/1 | China:Jiangsu |
| CCUG25004 | GCA_009663555.1 | 1976/1/1 | United Kingdom |
| NCTC11014 | GCA_900186945.1 | 1932 | status |
| CCUG25012 | GCA_028680055.1 | 1969 | United Kingdom |
| 17CS0503 | GCA_003024905.1 | 2017 | Germany |
| RCAD1086 | GCA_037861555.1 | 2020/8/26 | China:Sichuan, leshan |
| S63 | GCA_026427735.1 | 2020/5/20 | China |
| RCAD0422 | GCA_009690705.1 | 2017/4/17 | China:Guangdong |
| RCAD0421 | GCA_009690655.2 | 2017/4/17 | China:Guangdong |
| R-21 | GCA_024124965.1 | Jul-21 | China:Guangdong |
| RCAD0866 | GCA_033353655.2 | 2018 | China:Guangdong |
| PAT3 | GCA_042467385.1 | Apr-24 | China： Fujian |
| ZWRA063 | GCA_025907695.1 | 2019/12/14 | China:Jiangsu |
| RAf490 | GCA_046116525.1 | 2019/10/28 | China:Fujian |
| NN137 | GCA_025629605.1 | Sep-20 | China:Fujian |
| NN118 | GCA_025629665.1 | 2020/5 | China:Fujian |
| RCAD0416 | GCA_009670965.2 | 2017/5/5 | China:Sichuan, jinyang |
| 20190305E2-2 | GCA_019552325.1 | 2019/3/5 | China:Jiangsu |
| 20190121E1-3 | GCA_019552205.1 | 2019/1/21 | China:Jiangsu |
| WH9 | GCA_026016565.1 | 2016 | China:Henan |

| | | | |
|---|---|---|---|
| RCAD0133 | GCA_001670765.2 | 2011/3/26 | China:Chongzhou |
| JAGFUR01 | GCA_018474465.1 | 2019/5/2 | China:Jiangsu |
| 20190507E1-1 | GCA_021655845.1 | 2019/5/7 | China:Jiangsu |
| 59RAST107 | GCA_035666175.1 | 2023/5/2 | China:Fuyang |
| 20190509E1-1 | GCA_019802745.1 | 2019/5/9 | China:Jiangsu |
| RCAD0997 | GCA_051132245.1 | 2020/8/1 | China:Sichuan |
| LAVB01 | GCA_001263145.1 | 2011/12 | China:Hubei,jianli |
| VLIP01 | GCA_017614925.1 | 1969 | United Kingdom |
| HXb2 | GCA_002025185.1 | 2014/9/14 | China:Shanghai |
| SDAU-RA1 | GCA_052384455.1 | 2025/5/12 | China:Taian |
| JN1 | GCA_052049705.1 | 2024/12/1 | China:Shandong |
| RCAD0121 | GCA_001671145.1 | 2011/11/28 | China:Beijing |
| WJ4 | GCA_006385095.1 | 2000 | China:Jiangsu |
| CH3 | GCA_000734055.1 | 2014 | China |
| 20190604J2-1 | GCA_019552145.1 | 2019/6/4 | China:Jiangsu |
| RA-CH-1 | GCA_000295655.1 | 2014 | China:Sichuan |
| XG19 | GCA_018972085.1 | 2019/11/11 | China:Nanchang |
| 20160930RA1 | GCA_019802705.1 | 2016/9/30 | China:Jiangsu |
| RCAD0511 | GCA_030544805.1 | 2017/8/7 | China:Sichuan |
| RCAD0510 | GCA_030544785.1 | 2017/8/7 | China：Chongqing |
| RCAD0509 | GCA_030544765.1 | 2017/8/7 | China:Xizang,lasa |
| RA-LZ01 | GCA_009496935.1 | 2012/12/11 | China:Guangdong |
| RA-GD | GCA_000191565.1 | 2014 | China:Guangzhou |
| 20160930R-3 | GCA_019802785.1 | 2016/9/30 | China:Jiangsu |
| TLb2 | GCA_040498315.1 | 2013/6/11 | China:Shanghai |
| RAf950 | GCA_046101805.1 | 2024/1/9 | China:Fuzhou |
| RCAD0392 | GCA_015291805.1 | 1998/4/25 | China:Sichuan,jinyang |
| RA-NM | GCA_045348095.1 | 2020/7/20 | China:Jiangsu |
| RA-CH-2 | GCA_000331695.1 | 2014 | Generic |
| RCAD0134 | GCA_001670855.1 | 2011/4/10 | China:Jiangsu |
| RCAD0122 | GCA_001589345.2 | 2012/1/10 | China:Guangdong |
| ZWRA168 | GCA_025907525.1 | 2012/1/29 | China:Jiangsu |
| RCAD0183 | GCA_001670785.1 | 2014/7/8 | China:Guangdong |
| Yb2 | GCA_001077795.1 | 2015 | Generic |
| RA-YM | GCA_019297815.1 | May-01 | China:Hubei |
